# Editorial

**Published:** 1839-10-26

**Authors:** 


### THE MEDICAL EXAMINER.

PHILADELPHIA, OCTOBER 26, 1839.

The yellow fever has appeared at Martinique the present season, after a cessation of several years. Our correspondent, Dr. Rufz, writes that he finds that the most successful mode of treatment consists in repeated bleedings. This mode of treatment would seem at utter variance with the administration of quinine, in high doses, recommended by the physicians at New Orleans.

The discrepancy, however, is more apparent than real; for the New Orleans practice is designed to arrest the disease before the local lesions are developed, while the practice at Martinique is only directed to overcome the disease when the local lesions have fairly set in. This contradiction is, then, more apparent than real, and will explain the apparent discrepancy between the results of various methods of treatment in yellow fever.

